# Editorial: O_2_ and ROS Metabolisms in Photosynthetic Organisms

**DOI:** 10.3389/fpls.2020.618550

**Published:** 2020-11-23

**Authors:** Kentaro Ifuku, Anja Krieger-Liszkay, Ko Noguchi, Yuji Suzuki

**Affiliations:** ^1^Graduate School of Biostudies, Kyoto University, Kyoto, Japan; ^2^Université Paris-Saclay, CEA, CNRS, Institute for Integrative Biology of the Cell (I2BC), Gif-sur-Yvette, France; ^3^School of Life Sciences, Tokyo University of Pharmacy and Life Sciences, Tokyo, Japan; ^4^Faculty of Agriculture, Iwate University, Morioka, Japan

**Keywords:** photosynthesis, reactive oxygen species, electron transport (photosynthetic), redox regulation, oxidative stress

## Introduction

In photosynthesis, water is oxidized to O_2_, producing electrons to reduce CO_2_. During this process, reactive oxygen species (ROS) can be concomitantly produced. ROS cause oxidative damages if not scavenged appropriately. ROS also function as signaling molecules that change gene expression to alleviate stress conditions, reconstruct cellular functions, and allow acclimation to diverse environmental conditions. This Research Topic “O_2_ and ROS Metabolisms in Photosynthetic Organisms” comprises biochemical and molecular studies on mechanisms that suppress ROS production in photosynthetic organisms, as well as effects of ROS on photosynthesis in various species:

## Optimization of ATP Synthase c-Rings for Oxygenic Photosynthesis

Davis and Kramer examined the potential consequences of an altered c-ring stoichiometry of the chloroplast ATP synthase using their computational model of the photosynthetic light reactions, to which they added the possibility to vary the number of c-rings and thereby the *pmf* requirements to synthesize ATP. According to their results, the current c-ring stoichiometry is optimal; it minimizes detrimental side reactions producing ROS and allows high photosynthetic electron transport under a variety of environmental conditions.

## Identification of the Optimal Light Harvesting Antenna Size for High-Light Stress Mitigation in Plants

Wu et al. evaluated the effect of light growth intensities on mutant Camelina plants with diminished photosystem II (PSII) antenna sizes. Based on fluorescence yields, ROS and lipid peroxidation measurements, and biomass quantifications, the authors conclude that wild-type Camelina plants are well-suited for low light growth, whilst a reduction in PSII antenna size is beneficial at higher light intensities.

## New Light on Chloroplast Redox Regulation: Molecular Mechanism of Protein Thiol Oxidation

Yoshida et al. give a comprehensive and critical view of the light/dark redox regulation in chloroplasts. They highlight the recent evidence for regulatory disulfide bonds in oxidative pathways in the dark–a regulatory mechanism that has remained elusive since the discovery of the plant thioredoxin system. The newly uncovered “dark side” of chloroplast redox regulation provides an insight into how plants rest their photosynthetic activity at night.

## Singlet Oxygen Metabolism: From Genesis to Signaling

Dogra and Kim reviewed two distinct spatially separated ^1^O_2_ sensors and signaling pathways. The first pathway is triggered by oxidation of β-carotene or reactive electrophile species derived from lipids generated by ^1^O_2_ produced in PSII located in the grana region, while the second pathway involves the ^1^O_2_ sensor EXECUTER1 (EX1) protein located in non-appressed margins. They hypothesize a ^1^O_2_ generation site, likely tetrapyrroles, in margin PSII undergoing degradation after photoinhibition. EX1 is associated with the protease FtsH, and its proteolysis after oxidation is regarded as the essential step in initiating ^1^O_2_ signaling.

## Minimizing an Electron Flow to Molecular Oxygen in Photosynthetic Transfer Chain: An Evolutionary View

Kozuleva et al. describe in a comprehensive review the different sites of O_2_ reduction to the superoxide anion radical in respect to their importance with photosystem I (PSI) as the main site followed by the reduction of O_2_ by semiquinone in the plastoquinone pool. Thermodynamic and kinetics considerations are taken into account. Homologies and differences between anoxygenic and oxygenic photosynthetic complexes are discussed.

## Photorespiration Coupled with CO_2_ Assimilation Protects Photosystem I from Photoinhibition Under Moderate Peg-Induced Osmotic Stress in Rice

Photorespiration coupled with CO_2_ assimilation is thought to be a defense system against abiotic stress. Wada et al. showed the importance of photorespiration for the protection of PSI under osmotic stress, using transgenic rice plants with altered Rubisco content. Rubisco is thought to be the rate-limiting factor for both photorespiration and CO_2_ assimilation. Their results may contribute to improve abiotic stress tolerance.

## Primary Metabolite Responses to Oxidative Stress in Early-Senescing and Paraquat Resistant *Arabidopsis thaliana rcd1* (Radical-Induced Cell Death1)

The mutation of radical-induced cell death (RCD1) causes a resistant phenotype against methylviologen (MV), a herbicide generating ROS. *rcd1* mutant exerts tolerance to MV in an unknown way. Sipari et al. extensively analyzed metabolites by LC-MS and concluded that changes in primary metabolites cause the early senescing and MV-resistant *rcd1* phenotype. These findings will help to understand the ROS attenuation mechanism in plants, both in normal and stress conditions.

## Efficient Photosynthetic Functioning of *Arabidopsis thaliana* Through Electron Dissipation in Chloroplasts and Electron Export to Mitochondria Under Ammonium Nutrition

Long-term ammonium nutrition often disturbs growth and photosynthesis due to limited reductant utilization for ammonium assimilation. Podgórska et al. revealed that photosynthetic activity is supported by upregulation of cyclic electron flow around PSI, the plastid terminal oxidase, and the export of excess reductants from chloroplasts under long-term ammonium nutrition. Their results are important for understanding responses of photosynthetic regulation to environmental stresses.

## A Commonly Used Photosynthetic Inhibitor Fails to Block Electron Flow to Photosystem I in Intact Systems

Pharmacological approaches are commonly used to determine the sites of ROS generation by photosynthetic electron transport. Fitzpatrick et al. reported based on P700 absorption and MIMS measurements that the cytochrome *b*_6_*f* complex inhibitor 2,4-dinitrophenylether of iodonitrothymol (DNP-INT) is unable to completely block photosynthetic electron transport. These results ask for independent confirmation since DNP-INT is used as an alternative to 2,5-dibromo-6-isopropyl-3-methyl-1,4-benzoquinone (DBMIB), especially in spin-trapping assays used for ROS determination where DBMIB is known to interfere.

## Atmospheric CO_2_ Concentration and N Availability Affect the Balance of the Two Photosystems in Mature Leaves of Rice Plants Grown at a Free-Air CO_2_ Enrichment Site

Increasing atmospheric CO_2_ concentration ([CO_2_]) intensively affects photosynthesis and yield. The responses of photosynthesis to elevated [CO_2_] depends on nitrogen (N) availability. Ozaki et al. showed that elevated [CO_2_] and low N changed the balance of the two photosystems in leaves grown at a free-air CO_2_ enrichment experimental facility. This change may induce cyclic electron flow, increasing non-photochemical quenching to avoid photoinhibition.

## Author Contributions

All authors contributed to the final manuscript.

## Conflict of Interest

The authors declare that the research was conducted in the absence of any commercial or financial relationships that could be construed as a potential conflict of interest.

